# Why the mortality penalty of smoking is rising over time

**DOI:** 10.1093/pnasnexus/pgag280

**Published:** 2026-09-22

**Authors:** Ralph Lawton, Jennifer Beam Dowd, Jonathan Skinner, Ellen Meara

**Affiliations:** Interfaculty Initiative in Health Policy, Graduate School of Arts and Sciences, Harvard University, Cambridge, MA 02138, USA; Medical Scientist Training Program, Harvard Medical School, Boston, MA02115, USA; Leverhulme Center for Demographic Science, Nuffield Department of Population Health, Nuffield College, University of Oxford, Oxford OX1 1JD, United Kingdom; Department of Economics, Dartmouth College, Hanover, NH 03755, USA; National Bureau of Economic Research, Cambridge, MA 02138, USA; National Bureau of Economic Research, Cambridge, MA 02138, USA; Department of Health Policy and Management, Harvard T.H. Chan School of Public Health, Boston, MA 02115, USA

**Keywords:** smoking, mortality, aging

## Abstract

The adverse association between smoking and mortality is well known, yet estimated risks for smokers have risen markedly in recent decades. Explanations range from physiological impacts of changes in smoking intensity to shifts in the social characteristics of smokers. However, these hypotheses have rarely been tested. Using National Health Interview Survey data linked to the National Death Index through 2018, we confirm the mortality penalty associated with smoking increased by nearly 50% from 1987–1990 to 2012–2013, controlling for age. We show that this growing gradient between smokers and nonsmokers is driven largely by changes in the age distribution of smokers. This occurs even as more recent cohorts of smokers exhibit lower education, worse health, and greater psychological distress. These findings illustrate how evolving population composition can shape estimated mortality risks for exposures such as smoking and hold implications for understanding temporal changes in other risk factors like obesity.

Significance statement Smoking has large, adverse impacts on mortality and lifespan. However, substantial concern has surrounded potential increases over time in the association between smoking and mortality rates. We show that in data covering smoking behavior and mortality over nearly three decades, estimated increases in the mortality penalties associated with smoking are driven by changes in the age distribution of the population and of smokers. Over this period, the association between smoking and mortality at any given age has been stable.

## Introduction

Smoking remains one of the leading modifiable causes of death in the United States (1, 2). In recent years, concerns have been raised that the mortality risk associated with smoking may be increasing (3–6). Large cohort studies such as the Cancer Prevention Studies in the United States, or the British Doctors Study in the United Kingdom found that over the 20th century, the mortality risks associated with smoking have increased significantly (3, 7). More recent data in the United States suggest similar patterns have continued from the 1980s through 2006 (4).

There are several reasons why the mortality risk associated with smoking may have increased over time. The most direct mechanism would be an increase in the biological harm of smoking, through changes in cigarette smoke chemistry or the intensity of cigarette smoking. While this explanation is often mentioned, there is little evidence that it can explain time trends in the smoking–mortality association (3, 8). Alternatively, the characteristics of people who smoke may have changed over time, reflecting broader changes in public health, health behavior, and policy impacts (9, 10). People who smoke may be increasingly likely to live in places with other adverse health risks or participate in other behavior that raises mortality risk.

Since the release of the Surgeon General's Report in 1964, socioeconomic differences in smoking have widened. People without a high school degree make up an increasing share of smokers (11–13). People with low levels of education have faced increasing mortality risks in recent years, which may be reflected in the estimates of smoking mortality (9, 14). In this scenario, the direct biological risk of smoking has not necessarily increased, but smoking is more highly correlated with a wide range of underlying risks, both observed and unobserved.

Overall rates of current smoking in the United States have declined from 52% of males and 34% of females in 1965 to 13% of males and 10% of females in 2022. The timing of the peak of smoking differed by sex, with females both starting and quitting smoking later than males. Female smoking prevalence peaked in 1965–1966, while the prevalence for males peaked in the 1950s (15, 16). It is well-known that these changes in behavior across cohorts have substantially impacted aggregate cohort mortality (17, 18). In this paper, rather than assess the overall cohort burdens of smoking or the drivers of smoking behavior, we seek to examine whether or not the mortality penalty for individuals who smoke compared with those who do not smoke has changed over time. As smoking has become rarer and more socially stigmatized, its correlation with other health risk factors, such as lower education and mental illness, has strengthened (13, 19–22). These patterns are seen both in the United States and internationally (23).

Rapidly falling smoking rates in younger cohorts combined with an aging population (defined as a higher “proportion” of older people in the population) mean that the age distribution of smokers has become older over time. For example, 59% of ever smokers were over age 50 in 2013, compared with 42% in 1987. Given the exponential association between age and mortality, models that do not properly account for the shifting age distribution of smokers could yield biased estimates of an increasing smoking risk.

We test these potential mechanisms using nationally representative data from the 1987–2013 waves of the US National Health Interview Survey (NHIS) linked to death records through 2018, estimating how the association of smoking and all-cause mortality has changed over time. First, we successfully replicate estimates of increasing smoking penalties over this period. Next, we estimate the age-specific risk of mortality associated with smoking over three separate time periods to better account for shifts in the age composition of smokers over time. Finally, we estimate the changes in the relationship between smoking and other sociodemographic and health characteristics related to mortality, and whether these shifts impact smoking–mortality associations over time. We show that contrary to prevailing beliefs, the age-specific all-cause mortality penalties associated with smoking have changed little over nearly 30 years. This is true although smokers are increasingly less educated, report worse self-reported health, and report more psychological distress.

Taken together, these findings contribute to understanding how population change affects the mortality risks associated with smoking: a leading modifiable cause of death in the United States. These analyses particularly underscore the importance of carefully considering the intersection of age-specific health risks and changing age composition of those risks, especially with strong age-dependent outcomes like mortality.

## Results

### Trends over time in the smoking-associated mortality penalty

**Figure 1:**
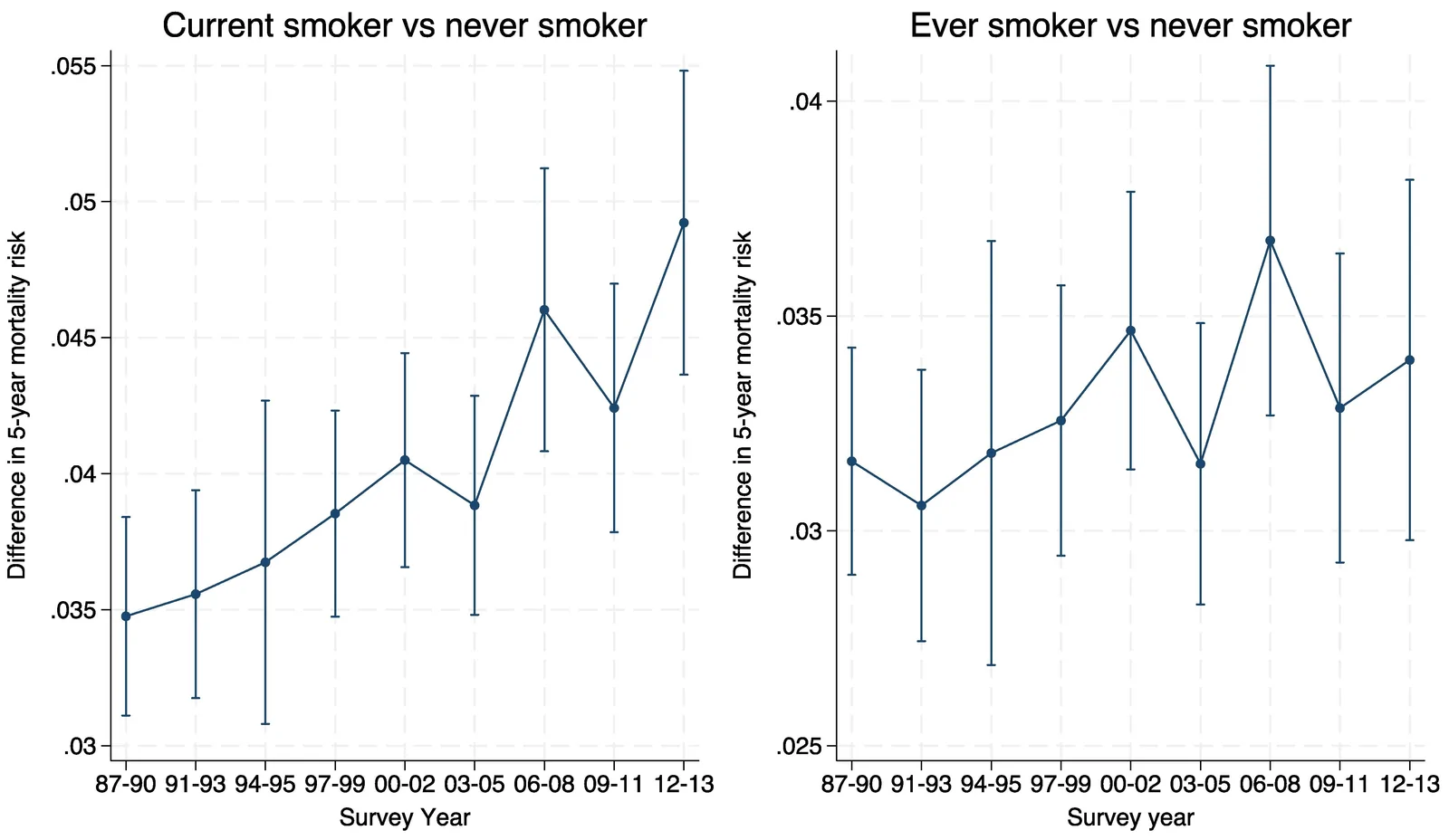
Trends in the smoking-associated 5-year mortality risk over time. Data are drawn from 25 to 84 year olds in the 1987–2013 waves of the National Health Interview Survey, who were followed to evaluate mortality for 5 years. Groups of three waves have been combined, and a weighted OLS model of 5-year mortality (died versus survived) is estimated in relation toa dummy for smoking behavior (either ever smoked 100 cigarettes or current smoker) and interactions for each year group controlling for age with a series of 5-year age dummies. The coefficient on smoking behavior and its 95% CI is plotted for each survey wave cluster. Thus, the figure shows the absolute difference in mortality rates associated with smoking behavior in each survey-year group. Over this period, the 5-year mortality risk difference for current vs. never smokers rose from 0.034 to 0.049 and rose from 0.032 to 0.034 (min: 0.031; max: 0.037) for ever vs. never smokers.

We begin by replicating documented increases over time in the smoking-associated mortality penalty found in other studies. Figure 1 shows the estimated 5-year mortality risk (died vs. survived) associated with smoking for different groups of NHIS survey years from 1987–2013 (with mortality data through 2018), controlling for age semi-parametrically using 5-year age bins in an ordinary least squares (OLS) model. We confirm an increase in estimated risk: comparing current smokers to never smokers, the probability of death increases by 44%, from 0.034 to 0.049. Comparing people who have smoked at least 100 cigarettes during their lifetime with those who have never smoked, we similarly find increases over time: from 0.031 to 0.037 at the peak, although risk in the final period is slightly lower than in the other recent periods. Similar patterns emerge when stratifying by sex or education, although the increases over time among men are somewhat more muted (Figs. S1 and S2).^a^

### Age-specific associations between smoking and mortality over time

**Figure 2:**
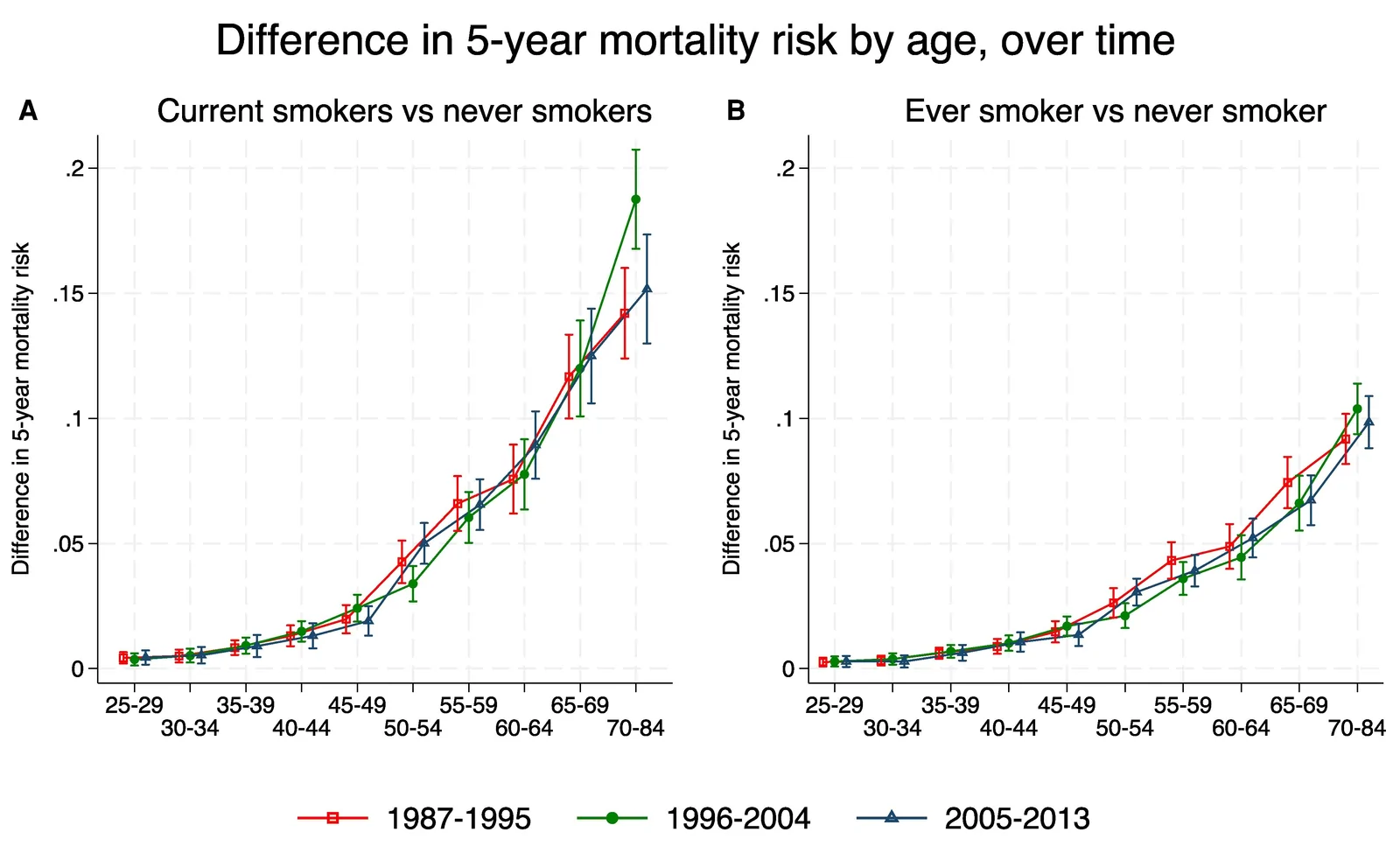
Difference in 5-year mortality risk by age, over time. The figure plots an age-specific estimate and 95% CI of the mortality risk associated with smoking in three time periods: 1987–1995, 1996–2004, and 2005–2013 using annual data from the NHIS. Panel A compares current smokers and never smokers, and Panel B compares ever smokers and never smokers. Data were restricted to respondents aged 25 years and older. Estimates were obtained from OLS regressions of 5-year mortality on age group, time period, and smoking status, and their (two-way and three-way) interactions. The figure displays the coefficients on smoker interacted with age group separately by time period. Time period markers in each age category are offset slightly for visibility.

To better account for changes in the prevalence of smoking across ages, we pool years of the NHIS into three time periods of about 8 years (1987–1995, 1996–2004, and 2005–2013—with 5-year mortality data spanning from 1987 to 2018) and estimate the age-specific association between smoking and mortality for each period. This approach contrasts with typical pooled methods that reflect an average of age-specific effects. Coefficients reflecting the difference in mortality probability between current smokers or ever smokers vs. never smokers at each age are plotted in Fig. 2, while absolute rates are plotted in Fig. S5. In each period, the smoking mortality risks increase nonlinearly at older ages and are low at younger ages. Smoking-associated mortality risks are also large relative to the base rates of mortality among nonsmokers. For example, in all periods, 5-year mortality risk among never smokers aged 60–64 was <0.05, compared to >0.10 for current smokers, a doubling of mortality risk for smokers at older ages.

Despite these large absolute risk differences, mortality risk associated with smoking at each age remains remarkably consistent across time periods. Notably, there is no age group for which the point estimate for the most recent period lies outside the 95% CI of the estimate for the earliest period. Conclusions are similar when stratifying the sample by sex (Fig. S6).^b^ Although age-specific differences between smokers and nonsmokers have changed little, mortality risk has improved for both smokers and nonsmokers over the study period (Fig. S5).

While we have shown that age-specific mortality risks have not changed over our study period, a related question asks, “To what extent can rising point-estimates of the smoking–mortality association be explained by changing age distributions?” There are several potential approaches to answer this question: across a series of decomposition approaches, we find that changing age distributions explain most of the increase in smoking-related mortality risk.

First, we model mortality in relation to smoking status reweighting the population to fix the age distribution to be the same as in 1987–1990 and controlling for age (in 5-year groups). Weights account for the impacts of an aging population on the contribution of each age group to pooled estimates. Age controls adjust for the impact of age on overall mortality risk if the age distribution of smokers and nonsmokers are different. Figures S3 and S4 show the unadjusted trends in panel A, where the smoking–mortality association increases >2-fold, then the independent impacts of age standardization via reweighting (panel B) and regression-based age controls (panel C), and their combined impacts in the bottom right (panel D). With this approach, relative to the increases shown in Fig. 1, the change in the current smoking coefficient from 1987–1990 to 2012–2013 is 63% lower and the ever- vs. never-smoking penalty actually declines slightly over time.

A second alternative uses the share of individuals in each age category in 2005–2013 and the 1987–1995 to 2005–2013 changes in the age-specific association between smoking and mortality to predict changes in the aggregate smoking–mortality coefficient.^c^ Comparing predicted to actual changes in the smoking–mortality parameter yields an estimate of the share of changes stemming from trends in the age distribution of smokers. This approach suggests that 82% of the increase in deaths among ever vs. never and 78% of the increase in deaths among current vs. never smokers are predicted by changes in the population age distribution.

Finally, we implement a formal decomposition of changes in the smoking–mortality relationship over time using the Juhn–Murphy–Pierce approach to decompose changes into changing demographic composition and changes in the coefficients on those demographic variables (24). In this decomposition comparing early (1987–1995) to more recent (2005–2013) periods, 60% of the increase in the mortality penalty for current vs. never smokers can be explained by the changing age of smokers, and 90% of the increase for ever vs. never smokers can be explained.

### Age-specific smoking risks and changing sociodemographic characteristics

Next, we examine how the sociodemographic and health characteristics of smokers have changed over time, and whether these changes impact the association between smoking and mortality over time.

**Figure 3:**
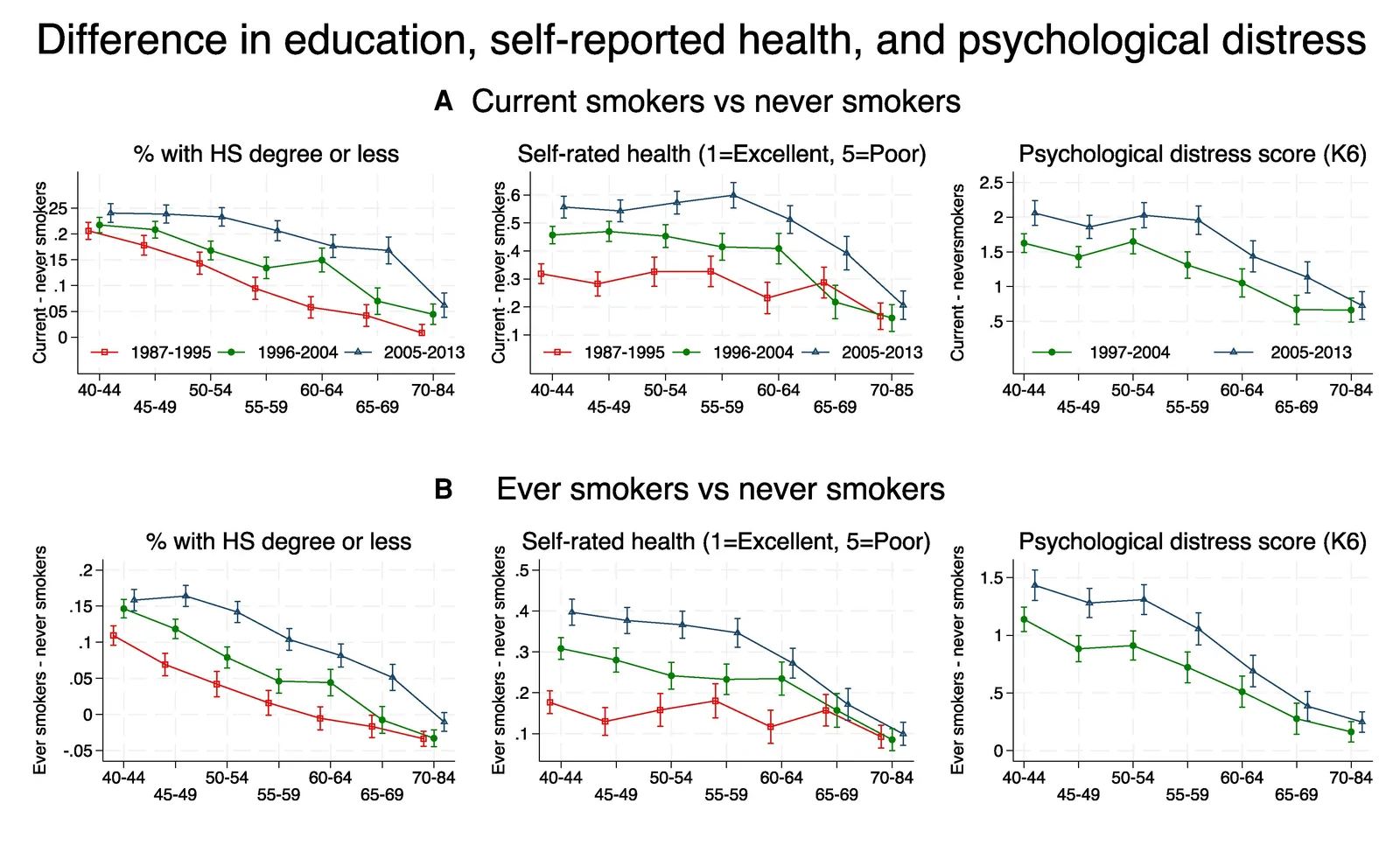
Age-specific smoking-associated education and health differences over time. Panel A compares current smokers with never smokers in each period, and Panel B compares ever smokers with never smokers. Higher Kessler-6 (K6) scores and self-rated health both reflect poorer health. Data are drawn from the 1987 to 2013 waves of the NHIS and is restricted to adults age 40+. Waves were combined into three larger categories of approximately seven waves each. Kessler-6 scores are only available 1997–2013. Estimates and 95% CIs are plotted based on a weighted OLS model of each respective outcome. The model includes indicators for 5-year age categories and a fully-saturated three-way interaction of age category, smoking behavior, and survey wave category.

Figure 3 shows differences in the age-specific levels of education, self-rated health, and psychological distress for current and ever smokers vs. never smokers across the three periods for adults over age 40.^d^ In more recent cohorts, gaps in self-rated health and psychological distress among smokers relative to never smokers at the same ages have widened. Smokers also have become disproportionately more likely to lack education beyond a high school degree over time, particularly in middle and older ages. For self-rated health and psychological distress, widening gaps comparing smokers and never smokers over time appear to be driven by adverse trends for smokers, with much more stable health and psychological distress among never smokers.

Given changes in the distribution of sociodemographic characteristics like educational attainment, or other potential comorbidities reflected in self-rated health, we examine whether these changes impact the age-specific mortality risk patterns we have observed. Figure S7 plots models that parallel Fig. 2 but adding controls for educational attainment and self-rated health. Even adjusting for these characteristics, age-specific smoking–mortality associations remain unchanged over time.

**Figure 4:**
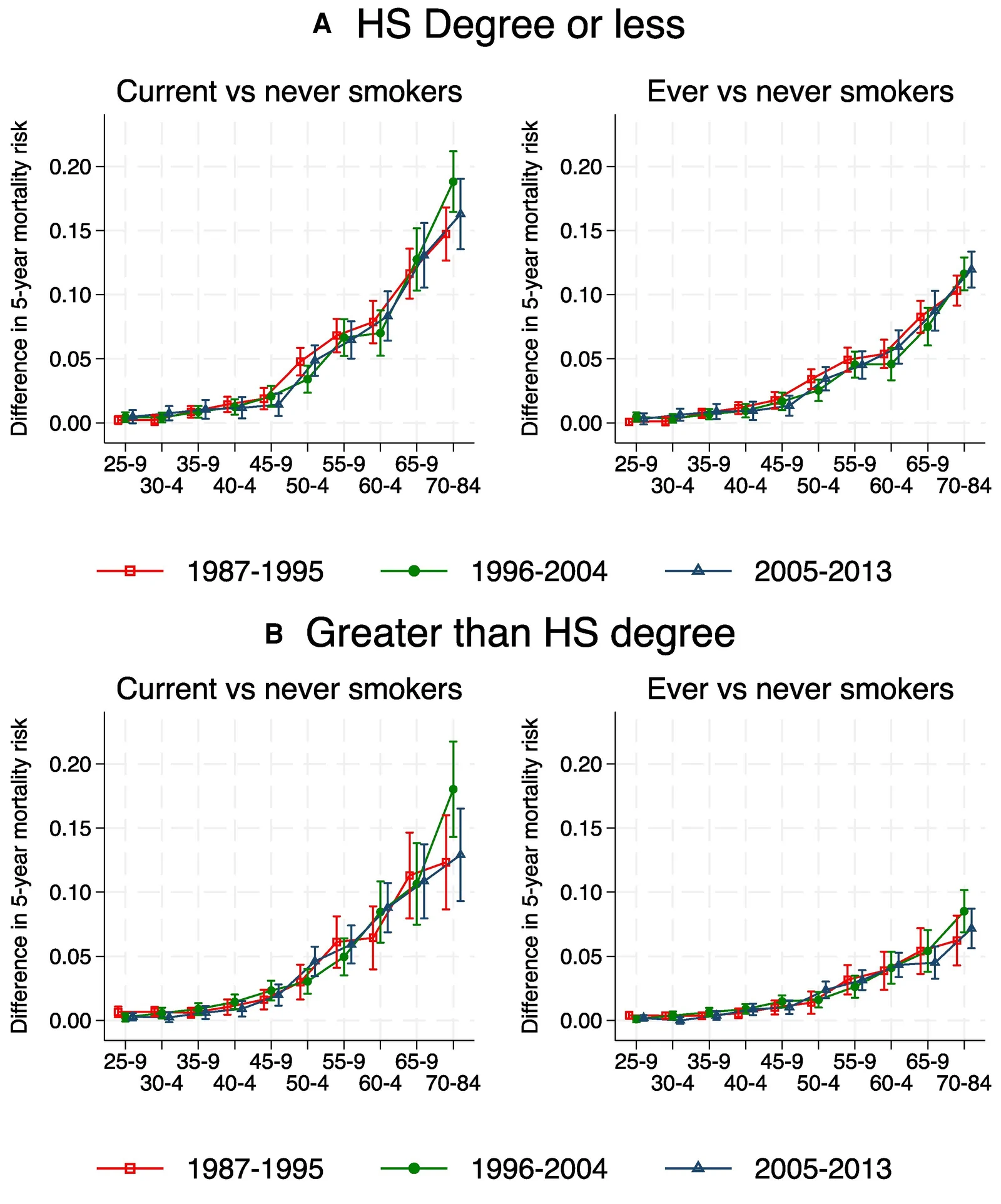
Age-specific smoking-associated mortality penalty over time, by education level. Panel A displays associations in each period for those with educational attainment of a high school degrees or less, and Panel B displays associations for those with educational attainment greater than a high school degree. Data are drawn from the 1987 to 2013 waves of the NHIS and are restricted to adults age 25+. Waves were combined into three larger categories of approximately seven waves each in order to raise statistical power. Estimates and 95% CIs are plotted based on a weighted OLS model of 5-year mortality as an outcome. The model includes indicators for 5-year age categories and a fully-saturated three-way interaction that includes age category, smoking behavior, and survey wave category. Estimated coefficients were used to produce the age-specific mortality risk difference between smokers and nonsmokers. Time period markers in each age category are offset slightly for visibility.

Examining differences in age-specific smoking risk by education rules out potential alternate explanations—that education-specific smoking risks may be changing in line with broader widening of education-mortality gaps (Fig. 4) (5, 14). Generally, the mortality penalty of current smoking is similar across education categories, although the penalty for ever smoking is somewhat lower in older age for those with more education. Overall, despite the changing educational composition of smokers, there is no evidence that the age-specific mortality penalties for current or former smokers within education categories have changed over time.

## Discussion

Contrary to well-known studies (3–6), we find evidence that the mortality risk associated with smoking has increased only minimally (if at all) during the last thirty years. Specifically, we show empirically that the age-specific estimates of the smoking–mortality relationship have been stable over time. After replicating estimates showing an increasing smoking–mortality association with conventional regression models, we reconcile increasing overall smoking–mortality associations over time with stable age-specific smoking mortality risks by accounting for the population aging of “smokers.”

We show this more formally in Appendix 1. Under commonly used regression estimators, the coefficient used to estimate smoking risk reflects an average of risks at different ages, weighted by (i) the size of the population at a given age as well as (ii) the variance of smoking at a given age. As the population has aged and smoking has declined in younger cohorts, conventional regression estimates of the association between smoking and mortality increasingly reflect the mortality risks of older smokers, which are substantially higher than those for younger smokers (Appendix 1). Notably, this implies that on their own, either reweighting to standardize the age distribution over time or utilizing simple age controls in a regression framework are not sufficient to estimate the relationship between smoking and mortality over time independent of age. Since the general population is aging and the distribution of smokers within the population is changing, estimates must account for both trends.

Our analysis and case study yield clear methodological implications that go beyond the examination of smoking: That simple age controls are not adequate for studying associations between risk factors and health over time where (i) there are heterogeneous effects by age and (ii) the distribution of a risk factor across age groups is changing. In our case, heterogeneous mortality effects by age are the primary concern, but this could in principle affect any dimension where health risks have substantial heterogeneity. For many conditions, mortality impacts are heterogeneous by age, and for behavioral changes such as diet, obesity, and physical activity, or even environmental exposures, there have been substantial changes in the distribution of risks across age groups over time.

Despite the stable association between smoking and mortality over time, we find that smokers are increasingly reporting lower levels of education, worse self-rated health, and more psychological distress relative to never smokers. Increasing negative selection on these characteristics (and potentially other unobserved characteristics) would be expected to increase the association of smoking and mortality over time, but models adjusting for education and self-rated health yield estimates that are nearly identical to our core findings (Fig. S7). The puzzle is at least partially resolved by noting that education and self-rated health are both significantly related to mortality risks, but the changes in these risk factors are small relative to the mortality risk associated with smoking. To take an example from our data, the mortality risk at age 55–59 associated with having high school or lower education is 2.8 percentage points (pp) and the likelihood that a smoker's education level is high school or lower rises over the study period by 11.8 percentage points. Thus, the changing educational composition of smokers would have increased the overall estimate of the smoking–mortality association by just 0.3pp (2.8pp*11.8pp), only a small fraction of the primary estimated coefficient of smoking on mortality for this age group (6.6pp). That is, while education is associated with mortality risk, the magnitude of the estimated impacts attributable to changing educational attainment among smokers is very small.

Using our age-specific estimates of the relationship between smoking and mortality, we show across several decomposition approaches that changes in the age distributions can explain the majority of the rise in aggregate estimates of the smoking–mortality association over time. We explain a larger share of the increasing mortality association with ever smoking than current smoking (82–103% explained, vs. 60–78% explained, respectively). This difference may reflect a more pronounced shift in the age distribution for ever smokers, especially at the oldest ages (Fig. S8). For example, the proportion of ever smokers aged 65 and over rose from 19% in 1987–1990 to 26% in 2012–2013, while among current smokers the proportion over 65 changed by only 1%, meaning that demographic corrections had less impact at older ages for current smokers. Additionally, for current smokers, the portion unexplained by shifting age composition could reflect longer durations of smoking for cohorts reaching older age in the 2010s compared with the 1980s due to younger average age of initiation (3, 25). Finally, it's possible that mortality selection—that later cohorts of current smokers were more likely to survive to their 60s and 70s than the past and carry that mortality risk with them—could account for some of the residual unexplained increases in risk.

Our findings also speak to a broader literature on the role of smoking in explaining increasing geographic inequalities and cohort patterns in mortality in the United States (5, 9, 17, 18, 26). While the association between county-level measures of smoking prevalence and life expectancy has been increasing, our findings suggest this is not driven by changes in the harms associated with smoking itself. More likely, stronger correlations between smoking prevalence and county-level mortality reflect widening spatial differences across counties with respect to their age structure and smoking rates; thus, some counties with relatively old populations may exhibit high rates of smoking, while other counties exhibit both younger populations and lower smoking rates. These variations are likely to grow over time as state policies and health risks grow more “clustered” over time (9, 26, 27).

Increasing midlife mortality among the least educated US adults has also received significant attention in recent years (9, 14, 28). Even among those with low education, we find that age-specific smoking mortality risks have not changed. In addition, controlling for changes in education and self-reported health over time does not impact estimates of age-specific smoking risks in the general population (Fig. S7). Taken together, this suggests that increases in the physiologic harms of smoking are unlikely to play an important role in rising age-adjusted midlife mortality rates for the least educated.

While we find that the age-specific relative and absolute penalty associated with smoking has not changed over time, mortality rates have declined for both smokers and nonsmokers (Fig. S5). This may reflect mortality gains associated with health behaviors other than smoking, or other improvements in medical technology shared by smokers and nonsmokers, such as improvements in treatment for cardiovascular disease and cancer over the time period (29–31).

Finally, these results have important methodological implications for projecting mortality due to smoking and other conditions. Prominent forecasts of smoking-attributable mortality, including from the Centers for Disease Control and Prevention (CDC), rest on relative risks of mortality for current and ever smokers (32). Smoking remains strongly associated with 5-year mortality—in most age groups, mortality rates for smokers are over twice that of never smokers. Nonetheless, estimating the mortality attributed to smoking and forecasting future deaths attributed to smoking should account for age-specific risks of smoking and changes in the age structure of smokers (4). Another implication of this work is that while the age structure of smokers matters substantially for the mortality impacts, other changes in the characteristics of smokers have had limited impacts on the age-specific associations between smoking and mortality.

Although this study examines smoking, the methodological implications for studying other risk factors that vary with age and across cohorts are broader. Mortality risk factors such as obesity, diet, or education may be changing across the age distribution over time, complicating our understanding of evolving risk.

## Data and methods

Data are drawn from the US NHIS (harmonized for the Integrated Public Use Microdata Series or IPUMS) from 1987–2013, linked to 5-year mortality data through 2018 from the National Death Index (33). We thus cover deaths occurring between 1987 and 2018. We exclude 1989 and 1996 survey waves because they omit respondent smoking information. The sample is comprised of adults aged 25–84 at the time of survey with complete information on smoking behavior, yielding a sample of 892,908 individuals. We analyze all-cause mortality to fully account for smoking risks across different physiologic systems and minimize misclassification from changes in diagnostic technology for specific causes of death (2).

We use two measures of smoking behavior: (i) “ever smokers” report they have smoked 100 cigarettes in their life, and (ii) “current smokers,” “ever smokers” who either self-identify as current smokers or report smoking cigarettes at least “some days” every week. All analyses compare either current or ever smokers with never smokers.

We use linear probability models to assess the relationship between smoking behavior and mortality over time and apply sample weights from the NHIS. (Alternative models using logistic regression or Cox proportional hazards models yield similar results).

First, we estimate the difference in 5-year mortality risk associated with smoking across time periods (1987/88/90, 1991–1993, 1994–1995, 1997–1999, 2000–2002, 2003–2005, 2006–2008, 2009–2011, 2012–2013) with a series of interactions between time period and smoking, controlling for age semi-parametrically with 5-year age bins (Fig. 1).

We build on this with a similar model estimating age-specific, rather than pooled, risks over time and divide the data into three time periods: 1987–1995, 1996–2004, and 2005–2013. We use 5-year age groups through age 69, but due to small sample sizes at older ages, we combine adults aged 70–84 into one group. Using two-way and three-way interactions for smoking status, age group, and time period, we estimate the smoking-associated mortality risk within each age group and time period and plot the age-specific coefficient on smoking for a given time period.

Given our age-specific findings, we decompose the role of changing age distributions in the aggregate changes in smoking-associated mortality risk. In one approach, we add several regression adjustments. We re-weight the sample to reflect the age distribution of the first time period and we include regression-based age adjustments and show the impacts of these adjustments on the estimated impact of smoking on mortality. This is similar to conventional age-adjustment approaches that fix the age distribution over time, but we also include controls for age as well as re-weight.

An alternative approach uses the change in regression-based estimates of the smoking–mortality associations in the 1987–1995 and 2005–2013 time periods (shown in Fig. 2), as well as the share of smokers in each age category in the 2005–2013 period, to predict changes in the aggregate association between smoking and mortality. We compare predicted changes in the aggregate coefficients with realized changes shown in Fig. 1. Multiplying the population shares in each age category by the change in the smoking coefficient in each age category predicts the amount the aggregate smoking–mortality association would have changed if only the age-specific smoking–mortality associations changed and the population distribution remained stable. The remainder of the aggregate increase in the smoking–mortality association can be attributed to changes in the distribution of smokers in each age category.

In a third approach, we decompose differences using the approach in Juhn–Murphy–Pierce, which decomposes changes in differences between groups into changes in the distribution of a covariate, changes in coefficients/associations, and changes in the distribution of residuals (24). We focus on age—and thus decompose the differences using a model that includes age as a series of age bins reflecting 5-year age groups and pooling ages 70–84 in order to match our age-specific models. Specifically, we decompose changes in the smoking–mortality association between the first (1987–1995) and last (2005–2013) time periods and emphasize the share of the changes explained by shifts in the age distributions.

Next, we examine how changes in education, self-rated health, and psychological distress have changed among smokers over this period. Education is coded as an indicator for greater than high school education (vs. high school or less). Self-rated health is assessed on a 1–5 scale, where 1 is excellent and 5 is poor, and psychological distress is assessed using the Kessler-6 (K6) (34). The K6 scale is only assessed from 1997 onwards. Empirically, these models closely reflect the model used to evaluate mortality. We estimated ordinary least squares regressions of each risk factor as a function of fully saturated interactions for smoking status, age group, and time period. We plot the coefficient on smoking in models of a given risk factor for each time period.

## Supplementary Material

pgag280_Supplementary_Data

## Data Availability

Data are publicly available from nhis.ipums.org (33). All replication code is available as a repository in the Harvard Dataverse: https://doi.org/10.7910/DVN/CIPNCK.

## Notes

a In particular, the ever vs. never smoker differences over time are more pronounced for women, with estimated risks increasing by 50%, while for men, it increases by <15%. This parallels findings by Thun et al. (3) and may in part reflect an earlier “peak” smoking for males than females.

b Mortality risks attributed to smoking by sex are indistinguishable in nearly all periods, with some exceptions. Among women aged 50–54 among current smokers, and ages 50–54 and 70–84 among ever smokers, mortality rates in the most recent period attributed to smoking were higher than in the earliest period. Among current smoker men aged 50–54, risks are lower in the middle period than in the earliest period. Overall, there is no evidence for systematic differences across time periods.

c While none of the age-specific changes in the mortality penalty associated with smoking are statistically significant comparing the first and last time periods, small age-specific changes may still lead to observed increases in the aggregate estimate. By predicting the changes in the aggregate estimate by changes in age-specific estimates, the potential share explained by changes in age-specific mortality risk can be examined.

d We emphasize differences in midlife in the interest of visibility—differences in these characteristics were primarily in midlife and older ages.
