# Supplementary material for: Why the mortality penalty of smoking is rising over time
Source: PNAS Nexus. 2026 Sep 22;5(9):pgag280. doi: 10.1093/pnasnexus/pgag280 (PMC13595352; doi:10.1093/pnasnexus/pgag280)
Supplement: Supplementary file 1 [file pgag280_supplementary_data.docx]

**Supplementary Material.**

Appendix 1. Econometric considerations in the implied weights when estimating mortality associated with smoking.

Consider a regression estimate of the relationship between smoking and mortality, conditional on age. For simplicity, let the age controls be specified flexibly, and assume that the only heterogeneity in the effects of smoking in the population emerge from different ages.

$$M_{i}=\beta_{0}+ \beta_{1}Smoking_{i}+ \beta_{2}Age_{i}+\varepsilon_{i}$$

where i denotes the individual (and we suppress the time index), M_i_ is 1 if the individual dies and 0 otherwise, and $\varepsilon_{i}$is the error term. We assume the true effect of smoking on mortality in the population varies with age; thus the overall coefficient on smoking will reflect a weighted average of age-specific effects. For simplicity, consider *K* age groups. Thus,

$$\beta_{1}= \sum_{k=1}^{K} w_{k}*\beta_{1k}$$

The weights $w_{k}$ will be proportional both to the size of a given age group and the variance of smoking within that age group:

$$w_{k}= \frac{p_{k}*V\left( Smoking \right|k)}{\sum_{k=1}^{K} p_{k}*V\left( Smoking \right|k)}$$

$$= \frac{p_{k}*p\left( Smoking=1 \right|k)*(1-p\left( Smoking=1 \right|k))}{\sum_{k=1}^{K} p_{k}*p\left( Smoking=1 \right|k)*(1-p\left( Smoking=1 \right|k))}$$

In the case of smoking, smoking prevalence is rarely greater than 0.5. Thus, $V\left( Smoking \right|k)$ is generally increasing in smoking prevalence within an age band. Two key features for our analysis are thus true. First, the weights applied to $\beta_{1k}$, the age-specific effect of smoking, increase as the population in an age group *k* increases. Second, in a context where smoking is getting relatively more common among the older population, $V\left( Smoking \right|k)$ is growing disproportionately larger for older age groups. As the population gets older, both forces add weight to $\beta_{1k}$ for greater *k.*

Conventional age-standardization looks to fix $p_{k}$ over time. However, if the distribution of smokers across age groups *k* is changing, then fixing $p_{k}$ does not actually fix $w_{k}$ over time, and thus $\beta_{1}$ would not be estimated to reflect the same age distribution in each period. Similarly, regression-based controls for age alone are not sufficient to estimate $\beta_{1}$ the same way in each time period.

**Appendix Figure 1**. **Trends in the smoking-associated 5-year mortality risk over time by sex.** Data are drawn from 25-84 year olds in the 1987-2013 waves of the National Health Interview Study, who were followed to evaluate mortality for 5 years. Groups of three waves have been combined, and within each group a weighted OLS model is estimated with a dummy for smoking behavior (either ever smoked 100 cigarettes, or current smoker) controlling for age with a series of 5-year age dummies. The coefficient on smoking behavior and 95% confidence interval estimated on smoking is plotted for survey wave cluster.**Appendix Figure 2**. **Trends in the smoking-associated 5-year mortality risk over time by education level.** Data are drawn from 25-84 year olds in the 1987-2013 waves of the National Health Interview Study, who were followed to evaluate mortality for 5 years. Groups of three waves have been combined, and within each group a weighted OLS model is estimated with a dummy for smoking behavior (either ever smoked 100 cigarettes, or current smoker) controlling for age with a series of 5-year age dummies. The coefficient on smoking behavior and 95% confidence interval estimated on smoking is plotted for survey wave cluster. Thus, figure 1 shows the absolute difference in mortality rates associated with smoking behavior in each survey-year group.

**Appendix Figure 3**. **Trends in the relative 5-year mortality risk associated with current smoking over time under different forms of age-adjustment.** Current smokers and never-smokers are compared. Data are drawn from 25-84 year olds in the 1987-2013 waves of the National Health Interview Study, who were followed to evaluate mortality for 5 years. Groups of three waves have been combined, and within each group a weighted OLS model is estimated with a dummy for smoking behavior (either ever smoked 100 cigarettes, or current smoker) controlling for age with a series of 5-year age dummies. In the first panel, unadjusted regression estimates of the relationship between smoking and 5-year mortality risk are displayed, simply using the sampling weights from the NHIS. In the second panel, these estimates are “age-adjusted” such that the NHIS-weighted population age distribution is held constant to match the first period. In the third panel, 5-year age dummies are used in a regression framework to control for age, using the NHIS sampling weights. Finally, a series of estimates over time are presented using both “age-adjustment” so that the population age distribution is fixed, and regression-based age controls are included.

**Appendix Figure 4.** **Trends in the relative 5-year mortality risk associated with ever smoking over time under different forms of age-adjustment.** Ever-smokers and never smokers are compared. Data are drawn from 25-84 year olds in the 1987-2013 waves of the National Health Interview Study, who were followed to evaluate mortality for 5 years. Groups of three waves have been combined, and within each group a weighted OLS model is estimated with a dummy for smoking behavior (either ever smoked 100 cigarettes, or current smoker) controlling for age with a series of 5-year age dummies. In the first panel, unadjusted regression estimates of the relationship between smoking and 5-year mortality risk are displayed, simply using the sampling weights from the NHIS. In the second panel, these estimates are “age-adjusted” such that the NHIS-weighted population age distribution is held constant to match the first period. In the third panel, 5-year age dummies are used in a regression framework to control for age, using the NHIS sampling weights. Finally, a series of estimates over time are presented using both “age-adjustment” so that the population age distribution is fixed, and regression-based age controls are included.

**Appendix Figure 5**: **Age-specific 5-year mortality estimates by smoking status, over time.** Data are drawn from the 1987 to 2013 waves of the NHIS and is restricted to adults age 25+. Waves were combined into three larger categories of approximately 7 waves. Estimates and confidence intervals are plotted from a weighted OLS model of 5-year mortality as a function of 5-year age category, smoking behavior, and time period (and their interactions). Coefficients yield the age-specific mortality risk of smokers and non-smokers in each period.

**Appendix Figure 6**: **Age-specific smoking-associated mortality penalty over time, by sex.** Data are drawn from the 1987 to 2013 waves of the NHIS and is restricted to adults age 25+. Waves were combined into three larger categories of approximately 7 waves each in order to raise statistical power. Estimates and confidence intervals are plotted from a weighted OLS model using 5-year mortality as a function of 5-year age category, smoking behavior, and time period (and their interactions). Coefficients yield the age-specific mortality risk difference between smokers and non-smokers in each period.

**Appendix Figure 7: Age-specific smoking-associated mortality penalty over time, controlling for education and self-rated health.** Data are drawn from the 1987 to 2013 waves of the NHIS and is restricted to adults age 25+. Waves were combined into three larger categories of approximately 7 waves each in order to raise statistical power. Estimates and confidence intervals are plotted from a weighted OLS model using 5-year mortality as an outcome. The model is specified with 5-year age categories, and regressions are estimated with a fully-saturated three-way interaction that includes age category, smoking behavior, and survey wave category. Further, they control for education (classified as <HS, =HS, >HS) and self-rated health flexibly with a series of dummies, which are interacted with age such that their effects are able to vary with age, but not over time. Coefficients from these estimates were then combined to produce the age-specific mortality risk difference between smokers and non-smokers.

**Appendix Figure 8: Age-distributions of ever- and current smokers over time.** Data are drawn from the 1987 to 2013 waves of the NHIS and is restricted to adults age 25+. Each bar plots the proportion of individuals who are ever smokers or current smokers who are each age, in 1987-90 and in 2012-13.

References:

[dataset] 1.	L. Blewett, *et al.*, IPUMS Health Surveys: National Health Interview Survey. https://doi.org/https://doi.org/10.18128/D070.V7.4. Deposited 2024.

2.	B. D. Lushniak, J. M. Samet, T. F. Pechacek, L. A. Norman, P. A. Taylor, The Health consequences of smoking—50 years of progress : a report of the Surgeon General.

3.	Carter Brian D., *et al.*, Smoking and Mortality — Beyond Established Causes. *New England Journal of Medicine* **372**, 631–640 (2015).

4.	Thun Michael J., *et al.*, 50-Year Trends in Smoking-Related Mortality in the United States. *New England Journal of Medicine* **368**, 351–364 (2013).

5.	N. Mehta, S. Preston, Continued Increases in the Relative Risk of Death From Smoking. *Am J Public Health* **102**, 2181–2186 (2012).

6.	D. M. Cutler, F. Lange, E. Meara, S. Richards-Shubik, C. J. Ruhm, Rising educational gradients in mortality: The role of behavioral risk factors. *Journal of Health Economics* **30**, 1174–1187 (2011).

7.	Female smoking death risk “has soared.” *BBC News* (2013).

8.	R. Doll, R. Peto, J. Boreham, I. Sutherland, Mortality in relation to smoking: 50 years’ observations on male British doctors. *BMJ* **328**, 1519 (2004).

9.	N. C. Institute (U.S.), *Risks Associated with Smoking Cigarettes with Low Machine-measured Yields of Tar and Nicotine* (U.S. Department of Health and Human Services, Public Health Service, National Institutes of Health, National Cancer Institute, 2001).

10.	B. K. Couillard, C. L. Foote, K. Gandhi, E. Meara, J. Skinner, Rising Geographic Disparities in US Mortality. *Journal of Economic Perspectives* **35**, 123–146 (2021).

11.	M. D. Stone, *et al.*, State and Sociodemographic Trends in US Cigarette Smoking With Future Projections. *JAMA Network Open* **8**, e256834 (2025).

12.	L. G. Escobedo, J. P. Peddicord, Smoking prevalence in US birth cohorts: the influence of gender and education. *Am J Public Health* **86**, 231–236 (1996).

13.	K. Giskes, *et al.*, Trends in smoking behaviour between 1985 and 2000 in nine European countries by education. *Journal of Epidemiology & Community Health* **59**, 395–401 (2005).

14.	N. J. Doogan, *et al.*, A growing geographic disparity: Rural and urban cigarette smoking trends in the United States. *Preventive Medicine* **104**, 79–85 (2017).

15.	E. R. Meara, S. Richards, D. M. Cutler, The Gap Gets Bigger: Changes In Mortality And Life Expectancy, By Education, 1981–2000. *Health Affairs* **27**, 350–360 (2008).

16.	Surveillance for Selected Tobacco-Use Behaviors -- United States, 1900-1994. Available at: https://www.cdc.gov/mmwr/preview/mmwrhtml/00033881.htm#00000793.htm [Accessed 10 March 2025].

17.	Achievements in Public Health, 1900-1999: Tobacco Use -- United States, 1900-1999. Available at: https://www.cdc.gov/mmwr/preview/mmwrhtml/mm4843a2.htm [Accessed 10 March 2025].

18.	S. H. Preston, H. Wang, Sex Mortality Differences in the United States: The Role of Cohort Smoking Patterns. *Demography* **43**, 631–646 (2006).

19.	H. Wang, S. H. Preston, Forecasting United States mortality using cohort smoking histories. *Proceedings of the National Academy of Sciences* **106**, 393–398 (2009).

20.	B. L. Cook, *et al.*, Trends in Smoking Among Adults With Mental Illness and Association Between Mental Health Treatment and Smoking Cessation. *JAMA* **311**, 172–182 (2014).

21.	J. P. Pierce, M. C. Fiore, T. E. Novotny, E. J. Hatziandreu, R. M. Davis, Trends in Cigarette Smoking in the United States: Educational Differences Are Increasing. *JAMA* **261**, 56–60 (1989).

22.	D. de Walque, Education, Information, and Smoking Decisions: Evidence from Smoking Histories in the United States, 1940–2000. *Journal of Human Resources* **45**, 682–717 (2010).

23.	J. M. Murphy, *et al.*, Cigarette Smoking in Relation to Depression: Historical Trends From the Stirling County Study. *AJP* **160**, 1663–1669 (2003).

24.	A. Teng, J. Atkinson, G. Disney, N. Wilson, T. Blakely, Changing smoking-mortality association over time and across social groups: National census-mortality cohort studies from 1981 to 2011. *Sci Rep* **7**, 11465 (2017).

25.	C. Juhn, K. Murphy, B. Pierce, “Accounting for the Slowdown in Black White Wage Convergence” in *Workers and Their Wages: Changing Patterns in the US*, (American Enterprise Institute, 1991).

26.	T. R. Holford, *et al.*, Patterns of Birth Cohort–Specific Smoking Histories, 1965–2009. *American Journal of Preventive Medicine* **46**, e31–e37 (2014).

27.	J. K. Montez, *et al.*, US State Policies, Politics, and Life Expectancy. *The Milbank Quarterly* **98**, 668–699 (2020).

28.	J. K. Montez, Deregulation, Devolution, and State Preemption Laws’ Impact on US Mortality Trends. *Am J Public Health* **107**, 1749–1750 (2017).

29.	A. Case, A. Deaton, Life expectancy in adulthood is falling for those without a BA degree, but as educational gaps have widened, racial gaps have narrowed. *Proceedings of the National Academy of Sciences* **118**, e2024777118 (2021).

30.	C. L. Avery, A. G. Howard, H. B. Nichols, Trends in US Cancer and Heart Disease Mortality, 1999–2018. *Circulation* **143**, 287–288 (2021).

31.	D. M. Cutler, *et al.*, A Satellite Account for Health in the United States. *American Economic Review* **112**, 494–533 (2022).

32.	T. Lu, *et al.*, Trends in the incidence, treatment, and survival of patients with lung cancer in the last four decades. *Cancer Management and Research* **11**, 943–953 (2019).

33.	Smoking-Attributable Mortality, Years of Potential Life Lost, and Productivity Losses --- United States, 2000--2004. Available at: https://www.cdc.gov/mmwr/preview/mmwrhtml/mm5745a3.htm [Accessed 10 March 2025].

34.	R. C. Kessler, *et al.*, Short screening scales to monitor population prevalences and trends in non-specific psychological distress. *Psychological Medicine* **32**, 959–976 (2002).
